# *Ex vivo* anti-malarial drugs sensitivity profile of *Plasmodium falciparum* field isolates from Burkina Faso five years after the national policy change

**DOI:** 10.1186/1475-2875-13-207

**Published:** 2014-05-31

**Authors:** Halidou Tinto, Léa N Bonkian, Louis A Nana, Isidore Yerbanga, Moussa Lingani, Adama Kazienga, Innocent Valéa, Hermann Sorgho, Hervé Kpoda, Tinga Robert Guiguemdé, Jean Bosco Ouédraogo, Petronella F Mens, Henk Schallig, Umberto D’Alessandro

**Affiliations:** 1 Unité de Recherche sur le Paludisme et Maladies Tropicales Négligées, Centre Muraz, Bobo-Dioulasso, Burkina Faso; 2 Institut de Recherche en Sciences de la Santé/Direction Régionale de l’Ouest (IRSS/DRO), Bobo-Dioulasso, Burkina Faso; 3 Clinical Research Unit of Nanoro (IRSS-CRUN), Nanoro, Burkina Faso; 4 Institut Supérieur des Sciences de la Santé (INSSA), Bobo Dioulasso, Burkina Faso; 5 Royal Tropical Institute/Koninklijk Instituut voor de Tropen (KIT), Amsterdam, The Netherlands; 6 Medical Research Council Unit, The Gambia, Disease Control & Elimination Theme, Fajara, The Gambia; 7 Prince Leopold Institute of Tropical Medicine, Antwerp, Belgium

## Abstract

**Background:**

The recent reports on the decreasing susceptibility of *Plasmodium falciparum* to artemisinin derivatives along the Thailand and Myanmar border are worrying. Indeed it may spread to India and then Africa, repeating the same pattern observed for chloroquine resistance. Therefore, it is essential to start monitoring *P. falciparum* sensitivity to artemisinin derivatives and its partner drugs in Africa. Efficacy of AL and ASAQ were tested by carrying out an *in vivo* drug efficacy test, with an *ex vivo* study against six anti-malarial drugs nested into it. Results of the latter are reported here.

**Methods:**

*Plasmodium falciparum ex-vivo* susceptibility to chloroquine (CQ), quinine (Q), lumefantrine (Lum), monodesethylamodiaquine (MDA), piperaquine (PPQ) and dihydroartemisinin (DHA) was investigated in children (6 months – 15 years) with a parasitaemia of at least ≥4,000/μl. The modified isotopic microtest technique was used. The results of cellular proliferation were analysed using ICEstimator software to determine the 50% inhibitory concentration (IC50) values.

**Results:**

DHA was the most potent among the 6 drugs tested, with IC50 values ranging from 0.8 nM to 0.9 nM (Geometric mean IC50 = 0.8 nM; 95% CI [0.8 - 0.9]). High IC50 values ranged between 0.8 nM to 166.1 nM were reported for lumefantrine (Geometric mean IC50 = 25.1 nM; 95% CI [22.4 - 28.2]). MDA and Q IC50s were significantly higher in CQ-resistant than in CQ-sensitive isolates (P = 0.0001). However, the opposite occurred for Lum and DHA (P < 0.001). No difference was observed for PPQ.

**Conclusion:**

Artemisinin derivatives are still very efficacious in Burkina Faso and DHA-PPQ seems a valuable alternative ACT. The high lumefantrine IC50 found in this study is worrying as it may indicate a decreasing efficacy of one of the first-line treatments. This should be further investigated and monitored over time with large *in vivo* and *ex vivo* studies that will include also plasma drug measurements.

## Background

Artemisinin-based combination therapy (ACT) has been deployed worldwide and is currently the only available effective treatment for falciparum malaria [1-4]. Nevertheless, the recent reports on the decreasing susceptibility of *Plasmodium falciparum* to artemisinin derivatives along the Thailand and Myanmar border [5-8] and more recently in Kenya [9] are worrying. Indeed, artemisinin-resistant malaria parasites at the border of Thailand and Myanmar may spread to India and then Africa, repeating the same pattern observed for chloroquine resistance [10]. It is, therefore, important to document the efficacy of currently used anti-malarials and provide early warnings that would allow an adequate response and the containment of resistance [11]. It is essential to start monitoring *P. falciparum* sensitivity to artemisinin derivatives and its partner drugs in Africa. This could be done by carrying out standard *in vivo* drug efficacy tests recommended by the World Health Organization (WHO). In addition, considering that there are no validated molecular markers related to artemisinins [6,8], *ex vivo* tests may have a role as several drugs, including those in a specific ACT, can be tested at the same time [12-15].

In Burkina Faso, a new malaria treatment policy was adopted in 2005 and was fully implemented in 2006; for uncomplicated falciparum malaria, artesunate-amodiaquine (ASAQ) or alternatively artemether-lumefantrine (AL) are recommended as first-line treatments, whereas quinine is recommended for severe malaria [16]. Efficacy of AL and ASAQ were tested by carrying out an *in vivo* drug efficacy test, with an *ex vivo* study against six anti-malarial drugs nested into it. Results of the latter are reported here.

## Methods

### *Plasmodium falciparum* field isolates

The study was carried out from December 2008 to December 2010 in Bobo-Dioulasso situated at 365 km from Ouagadougou. The rainy season occurs from June to October (average rainfall: 1,000 mm/year; mean temperature >25°C) and is followed by a cold dry season from November to February (minimum temperature 15°C) and a hot dry season from March to May. Malaria transmission is seasonal, from June to December. The commonest vectors are *Anopheles gambiae s.s.*, *Anopheles funestus* and *Anopheles arabiensis*, and *P. falciparum* is the predominant malaria parasite [17]. All children (6 months-15 years) with fever (axillary temperature ≥ 37.5°C) or history of fever in the last 24 hours were screened for malaria infection. Children weighing 5 kg or more with a *P. falciparum* mono-infection at a density between 4,000 and 200,000/μl and haemoglobin > 7 g/dL were included in the study if their parent/guardian provided the informed consent. Venous blood (5 – 10 ml) was collected in a tube coated with EDTA (Turumo, Escap, Belgium) for *ex vivo* tests. This study was reviewed and approved by the Institutional Ethics Committee of Center Muraz, and was registered at ClinicalTrials.gov (ID: NCT00808951).

### Drugs

The drugs tested were from the following sources: chloroquine diphosphate (Sigma Aldrich, St. Louis, USA), quinine hydrochloride (Sigma Aldrich, St. Louis, USA), lumefantrine (Novartis Pharma, Basel, Switzerland) piperaquine phosphate (Sigma Tau., Rome, Italy), the active metabolite of artemisinin, dihydroartemisinin (Sigma Tau., Rome, Italy) and the active metabolite of amodiaquine, monodeshethylamodiaquine obtained from the World Health Organization (WHO/TDR, Geneva, Switzerland). The stock solutions of chloroquine and monodesethylamodiaquine were prepared in sterile distilled water, in methanol for quinine and dihydroartemisinin, in ethanol for lumefantrine and in lactic acid for piperaquine. A three-fold serial dilution of stock solutions was made. The final concentrations ranged from 12.5 nM to 3200 nM for chloroquine, 7.5 nM to 1920 nM for monodesethylamodiaquine, 13.02 nM to 3333 nM for quinine, 1.25 nM to 320 nM for lumefantrine, 6.25 nM to 1600 nM for piperaquine and 0.25 nM to 64 nM for dihydroartemisinine. The distribution in the plates was done at 50 μl per well.

### *Ex vivo* assay

Drug sensitivity tests were performed within 24 hours of bleeding, without culture adaptation. Blood samples were washed three times in RPMI 1640 medium (Sigma Aldrich, St. Louis, USA). The modified isotopic microtest technique [14] was used. The complete RPMI 1640 medium, i.e. supplemented with 10% of human serum type AB (obtained from the blood bank of Souro Sanon University hospital, Bobo-Dioulasso, Burkina Faso), gentamicin (10 μg/ml) and tritiated hypoxanthine (Amersham, little Chalfont, UK), was used for parasites cultivation. Infected erythrocytes were suspended in this medium at a haematocrit of 1.5% and an initial parasitaemia between 0.1% and 0.5%. This suspension was then added in quantities of 200 μl per well to the plates containing the drugs. These were incubated for 48 hours at 37°C in 5% CO_2._ After the incubation period, plates were frozen and thawed to lyse the blood cells. Cultures were then harvested (Harvester Unifilter 96, Packard) on fibre glass paper (reader plates Unifilter 96 Perkin Elmer). The strips obtained were dried and transferred to a plastic bag containing 30 μl of scintillation fluid (Perkin Elmer Betaplate Scint, Wallac). The incorporation of tritiated hypoxanthine was measured using a scintillation Beta counter (Perkin Elmer Wallac MicroBeta Trilux, Turku, Finland).

### Data analysis and statistical methods

For parasites growth assays, the results of cellular proliferation were expressed as counts per minute and analysed using ICEstimator software to determine the 50% inhibitory concentration (IC_50_) values [18]. The IC_50_ is defined as the drug concentration able to inhibit 50% of the uptake of ^3^H hypoxanthine as compared to that measured in drug-free control wells. The threshold IC_50_ for *ex vivo* resistance was defined at ≥ 100 nM for chloroquine, ≥ 60 nM for monodesethylamodiaquine and ≥ 800 nM for quinine [19,20]. The thresholds for lumefantrine, piperaquine and dihydroartemisinin are not well established yet. Data were entered in Excel version 97 and analysed using Stata version 8.0. Results were expressed as geometric mean IC_50_ values and the 95% confidence intervals were computed. Correlation of the IC_50_ values for different drugs (2 by 2) was calculated using Spearman rank-order correlation test. The activity of monodesethylamodiaquine, quinine piperaquine, lumefantrine and dihydroartemisinin against chloroquine-resistant isolates and chloroquine-sensitive isolates were compared using the IC_50_ geometric means (GM). A p value <0.05 was considered statistically significant.

## Results

**Figure 1:**
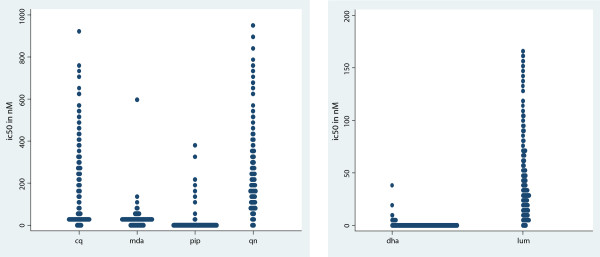
**Ditribution of IC**_**50s **_**values of *P. falciparum ex vivo *** **susceptibility against chloroquine (cq), monodestylamodiaquine (mda), piperaquine (pip); quinine (qn); dihydroartemisinin (dha) and lumefantrine (lum).**

*Ex vivo* susceptibility of *P. falciparum* isolates was tested for 440 samples. The culture success rate (interpretable tests) and the IC_50_ geometric means of the 6 drugs tested are summarized in Table 1. The average culture success rate was around 85% (range: 79.3% - 86.8%). Out of 382 samples successfully tested against chloroquine (Geometric mean IC_50_ = 69.2 nM; 95% CI [60.6 - 79.1]) and quinine (Geometric mean IC_50_ = 162.1 nM; 95% CI [148.3 - 177.3]), 161 (42.1%) were resistant to chloroquine while only 4 (1%) were resistant to quinine. Out of 377 samples successfully tested against monodeshethylamodiaquine (Geometric mean IC_50_ = 19.3 nM; 95% CI [18.0 - 20.6]), 24 (6.4%) were resistant. The IC_50_ values for lumefantrine ranged between 0.8 nM to 166.1 nM (Geometric mean IC_50_ = 25.1 nM; 95% CI [22.4 - 28.2]) and that for piperaquine from 0.8 nM to 375.2 nM (Geometric mean IC_50_ = 6.3 nM; 95% CI [5.9 - 6.8]). Dihydroartemisinin was the most potent among the six drugs tested, with IC_50_ values ranging from 0.8 nM to 0.9 nM (Geometric mean IC_50_ = 0.8 nM; 95% CI [0.8 - 0.9]). However, three isolates had much higher IC_50_, i.e. 19 nM, 21 nM and 38 nM (Figure 1).

**Table 1:** ***Ex vivo *** **susceptibility of *Plasmodium falciparum *** **isolates against chloroquine, monodeshethylamodiaquine, quinine, lumefantrine and dihydroartemisinin**

	**Culture success rate % (n/N)**	**IC50 mean (nM) [95% CI]**	**Range (nM)**		**Resistant isolates (%)**
			**Minimum**	**Maximum**	
Chloroquine	86.8 (382/440)	69.2 [60.6 – 79.1]	8.3	595.9	161 (42.1)
Quinine	86.8 (382/440)	162.1 [148.3 – 177.3]	10.2	950.3	4 (1.0)
Monodesethylamodiaquine	85.7 (377/440)	19.3 [18.0 – 20.6]	0.8	595.9	24 (6.4)
Lumefantrine	86.8 (382/440)	25.1 [22.4 – 28.2]	0.8	166.1	NA
Piperaquine	79.3 (349/440)	6.3 [5.9 – 6.8]	0.8	375.2	NA
Dihydroartemisinin	86.5 (381/440)	0.8 [0.8 – 0.9]	0.1	38.8	NA

Resistance threshold defined for chloroquine at 100 nM, quinine at 800 nM and monodeshethylamodiaquine at 60 nM; NA: Non applicable as the threshold is not defined yet.

The mean IC_50_ of the tested drugs were analysed by the parasites’ susceptibility to chloroquine, i.e. chloroquine-resistant (IC50 ≥ 100 nM) against chloroquine-sensitive isolates (IC50 < 100 nM). Monodesethylamodiaquine and quinine IC_50_ mean values were significantly higher in chloroquine-resistant than in chloroquine-sensitive isolates (P = 0.0001) (Table 2). However, the opposite occurred for lumefantrine and dihydroartemisinin; their IC_50_ mean values were significantly higher in chloroquine-sensitive than in chloroquine-resistant isolates (P ≤ 0.001). No difference was observed for piperaquine (P = 0.382).

**Table 2:** ***Ex vivo *** **IC**_**50 **_**of *Plasmodium falciparum *** **isolates against monodeshethylamodiaquine, quinine, lumefantrine, piperaquine and dihydroartemisinin by chloroquine susceptibility**

**Drug**	**Chloroquine-resistant isolates (n = 161)**	**Chloroquine-sensitive isolates (n = 221)**	**P value**
Chloroquine	289.8 [269.2 – 312.0)]	24.4 (22.8 – 26.1)	0.0001
Monodesethylamodiaquine	33.8 [31.4 – 36.4]	12.8 [12.0 – 13.6]	0.0001
Quinine	249.7 [223.3 – 279.1]	120.4 [107.2 – 135.2]	0.0001
Lumefantrine	21.9 [18.8 – 25.5]	27.8 [23.5 – 32.8]	0.0006
Piperaquine	6.5 [5.7 – 7.3]	6.1 [5.6 – 6.7]	0.382
Dihydroartemisinin	0.7 [0.6 – 0.8]	0.9 [0.8 – 1.0]	0.001

Cross-resistance between the six drugs is summarized in Table 3. A significant positive correlation (by ascending order) was found between monodeshetlyamodiaquine-piperaquine (r = 0.14; P = 0.008), dihydroartemisinin-quinine (r = 0.15; P = 0.002), dihydroartemisinin- piperaquine (r = 0.27; P < 0.0001), dihydroartemisinin-lumefantrine (r = 0.30; P < 0.0001), quinine - lumefantrine (r = 0.32; P < 0.0001), chloroquine-quinine (r = 0.51; P < 0.0001), monodeshetlyamodiaquine-quinine (r = 0.52; P < 0.0001) and chloroquine-monodeshetylamodiaquine (r = 0.86; P < 0.0001). For chloroquine-lumefantrine (r = - 0.10; P = 0.03) and monodeshetylamodiaquine-lumefantrine (r = - 0.11; P = 0.02) the correlation was significant but negative while for the other pair-wise comparisons no significant correlation was found.

**Table 3:** **Pairwise comparison of *ex vivo *** **IC**_**50 **_**values**

**Drug pairs**	r*	***P - value***
Chloroquine - Dihydroartemisinin	-0.08	0.09
Chloroquine - Monodestylamodiaquine	0.86	< 0.0001
Chloroquine – quinine	0.51	< 0.0001
Chloroquine - Piperaquine	0.02	0.63
Chloroquine - Lumefantrine	-0.10	0.03
Dihydroartemisinin- Monodestylamodiaquine	0.01	0.77
Dihydroartemisinin – Quinine	0.15	0.002
Dihydroartemisinin- Piperaquine	0.27	< 0.0001
Dihydroartemisinin - Lumefantrine	0.30	< 0.0001
Monodestylamodiaquine – Quinine	0.52	< 0.0001
Monodestylamodiaquine - Piperaquine	0.14	0.008
Monodestylamodiaquine - Lumefantrine	-0.11	0.025
Quinine - Piperaquine	0.07	0.16
Quinine – Lumefantrine	0.32	< 0.0001
Piperaquine - Lumefantrine	0.04	0.35

*Spearman’s rank-order correlation coefficient (r).

## Discussion

Since the policy change in 2005 in Burkina Faso, several studies on the therapeutic efficacy of both ASAQ and AL were carried out [21-25]. Nevertheless, the *ex vivo* susceptibility of *P. falciparum* to the different components of ACT had never been tested and this is the first study out of this kind. The prevalence of chloroquine resistant isolates (CQR) was higher than that against other drugs but lower than that reported in the same area in 2006 and estimated at 50% (Lea Bonkian, personal communication), suggesting that CQ resistance may be decreasing, possibly following the implementation of the new anti-malarial drug policy based on ACT. This is plausible when considering that a similar phenomenon has been observed in Malawi where, nine years after the withdrawn of CQ and its replacement with sulphadoxine-pyrimethamine, no CQR was found [26].

Despite withdrawal of chloroquine, CQR could persist due to the use of treatments with similar chemical structure, resulting in a strong selective pressure [27]. The positive correlation between *ex vivo* IC_50_ values of CQ and MDA or quinine and between quinine and AQ indicate cross-resistance and may explain the still high prevalence of CQR found in this study. Such cross-resistance is not surprising as it has already been reported by several studies [20,28,29]. Nevertheless, the relationship between CQ and AQ efficacy is not a straightforward one as AQ may still be effective where CQ resistance is high [1,30-32]. This seems confirmed by the low prevalence of AQ resistant isolates as determined by our *ex vivo* test. Nevertheless, almost half of the isolates had AQ IC_50_ values higher than 20 nM, a relatively high figure when considering that in Cameroun most isolates of recurrent infection after AQ treatment had IC_50_ ranging between 25.6 and 115 nM, indicating that the threshold for AQ resistance might be lower than the standard value of ≥ 60 nM [19]. This raises the issue of defining appropriate drugs’ *ex vivo* IC_50_ thresholds able to predict *in vivo* outcomes [19,27,33,34].

In the new malaria treatment policy adopted in Burkina Faso, quinine is still the recommended treatment for severe malaria and for any treatment failure after administration of ASAQ or AL [16]. Only 4 isolates were found to be resistant to quinine, an extremely low prevalence when considering the potentially frequent use of this drug and cross resistance with CQ. Lowering the threshold for resistance to 600 nM [28,35,36], increased the number of isolates classified as resistant but their prevalence remains low, i.e. 4% (17/382). Therefore, quinine can still be considered effective in Burkina Faso though recent reports of an increasing number of patients with recurrent infection after ACT treatment [21,24] may result in the frequent use of quinine as rescue treatment and higher drug pressure. There is the need of regularly monitoring the susceptibility of local isolates to quinine for the early detection of quinine resistance.

The resistance threshold for lumefantrine is not established yet but the mean IC_50_ (25.1 nM) found in this study seems high compared to that (9.8 nM) reported by a study carried out in Senegal at approximately the same period [37]. Similarly, in Cameroon the mean IC_50_ for lumefantrine was 11.9 nM in 1997 and 9.57 nM in 2003 [38]. Therefore, high lumefantrine IC_50_ may indicate a decreasing susceptibility of local isolates to this drug, though baseline data are not available, possibly explained by the high AL use and hence drug pressure in this urban area. Indeed, though Burkina Faso has adopted two types of ACT as first-line treatments, ASAQ is mainly used in rural areas while AL in towns, including Bobo-Dioulasso where this study was carried out. This is also confirmed by informal discussions with local health practitioners who stated that they mostly prescribe AL for uncomplicated malaria. However, such hypotheses need to be confirmed by a well-planned survey. The high lumefantrine IC_50_ could also be due to technical problems related to the execution of the *ex vivo* test. Indeed, lumefantrine is an amino alcohol and the drugs in this class are not easily soluble, a characteristic that may compromise the reproducibility of *ex vivo* test results [39]. Nevertheless, the use of the ethanol as solvent in this study should have addressed this problem so that a major bias related to the lumefantrine solubility is unlikely.

Dihydroartemisinin-piperaquine is one of the most recent ACT submitted for prequalification to the WHO [40] and represents an additional ACT for endemic countries, including Burkina Faso. Mean piperaquine IC_50_ was extremely low, only two isolates had values above 200 nM, and similar to that observed in Uganda [41]. These results contrast with those found in Cameroon and Kenya, where the mean piperaquine IC_50_ was 39 nM and 50 nM, respectively [42,43]. In addition, there was no correlation between piperaquine and chloroquine, lumefantrine and quinine IC_50_s, a weak correlation with MDA, and piperaquine was equally active on both CQ sensitive and resistant isolates. All these elements support the use of dihydroartemisinin-piperaquine as an alternative ACT in Burkina Faso.

The mean dihydroartemisinin IC_50_ was extremely low, indicating high susceptibility of local isolates, and in accordance with other studies carried out in sub-Saharan Africa [36,38,44]. In addition, dihydroartemisinin IC_50_ was not correlated to that of MDA while the correlation with CQ IC_50_ was a negative one, also shown by its higher activity against CQ resistant isolates as compared to sensitive ones. This is reassuring when considering the threat represented by the emergence of artemisinin resistance in South-East Asia where, besides a delay in parasite clearance, the *ex vivo* sensitivity of *P. falciparum* to artemisinin derivatives has declined substantially over the last few years [2,4-6,8,45,46], reaching in some regions mean IC_50_ values as high 21.2 nM for dihydroartemisinin and 16.3 nM for artesunate [47]. Nevertheless, the interpretation of this results should also consider the weaknesses of the standard *ex vivo* tests, e.g. the 3H hypoxantine technique, in detecting artemisinin derivatives resistance [48]. Though in sub-Saharan Africa the *ex vivo* efficacy of artemisinin derivatives seems high, declining *in vivo* responsiveness of *P. falciparum* infections to ACT has been observed in Kenya [9].

## Conclusion

In conclusion, this study confirms that artemisinin derivatives are still very efficacious and dihydroartemisinin-piperaquine seems a valuable alternative ACT in Burkina Faso. Similarly, both quinine and amodiaquine had a good sensitivity profile. The high lumefantrine IC_50_ found in this study is worrying as it may indicate a decreasing efficacy of one the first line treatments. This should be further investigated and monitored over time with large *in vivo* and *ex vivo* studies that will include also plasma drug measurements.

## Competing interest

The authors declare that they have no competing interests.

## Authors’ contribution

The study was conceived by HT, UDA, PFM and HS in the framework of the MALACTRES project. It was conducted in the field by HT, NLB, LAN, IY, ML IV, HS and HK and supervised by JBO and TRG. Data analysis was done by AK, IV and HT. The manuscript was drafted by HT and UDA. All authors read and approved the final manuscript.
